# The effects of soil freeze–thaw processes on water and salt migrations in the western Songnen Plain, China

**DOI:** 10.1038/s41598-021-83294-x

**Published:** 2021-02-16

**Authors:** Yan Qin, Yufeng Bai, Guoshuang Chen, Yunjiang Liang, Xiaoyu Li, Bolong Wen, Xinrui Lu, Xiujun Li

**Affiliations:** 1 Northeast Institute of Geography and Agroecology, Chinese Academy of Sciences, Changchun, 130102 People’s Republic of China; 2 University of Chinese Academy of Sciences, Beijing, 100049 People’s Republic of China; 3 College of Agriculture, Yanbian University, Yanji, 133002 Jilin People’s Republic of China

**Keywords:** Ecology, Ecology, Grassland ecology, Wetlands ecology, Environmental sciences, Environmental impact

## Abstract

Seasonally freeze-thaw (*FT*) processes affect soil salinisation in cold and arid regions. Therefore, understanding the mechanisms behind soil salinisation during winter and spring is crucial for management strategies effectively alleviating this. This study aimed to explore the soil *FT* characteristics and their influences on soil water and salt migrations to clarify the underlying mechanism of the springtime soil salinisation in the western Songnen Plain, China. The spatiotemporal distributions of soil water and salt, frozen depths and soil temperatures were examined at depths of 0–200 cm in three typical landscapes (farmland, *Leymus chinensis* (*Trin*.) *Tzvel* (*LT*) grassland and alkali-spot (*AS*) land) from October 2015 to June 2016. Results indicated that the strongest freezing process occurred in *AS* land, which was characterised by the deepest frost depth (165 cm) and highest freezing rate (3.58 cm/d), followed by *LT* grassland, and then farmland. The freeze-induced upward redistribution and enrichment of soil water and salt caused the rise and expansion of the soil salification layer, which was the main source of explosive accumulations of surface salt in springtime. Therefore, the *FT* processes contributed to the surface soil salinisation and alkalisation. Landscapes also affected soil water and salt migrations during *FT* processes, with the trend being *AS* land > *LT* grassland > farmland.

## Introduction

Soil freeze–thaw (*FT*) cycles is a constantly repeating process of material exchange and energy transfer that occurs in the surface soil and also extends downward into the deeper soil layers^1,2^. Understanding these cycles in cold regions is crucial owing to their vital roles in agricultural and ecological environments, because they have profound influences on soil water distribution, heat balance, and spring farming and plant germination^3^. To enhance the collective knowledge of soil *FT* processes, a more comprehensive monitoring regime of hydrological variables, such as soil water content (*SWC*), soil salt content, soil temperature, frost depth, groundwater levels, are required. In turn, *FT* cycles also influence these hydrological variables. Several researchers examined the interactions between hydrological variables and *FT* cycles in cold and arid regions. For example, the analysis by Bing et al. (2015) indicated that *FT* processes acted as a controlling mechanism for the redistribution of soil water and salt through analyzing the factors that influence soil water and salt migrated behaviours in the Qinghai–Tibet Plateau of China^4^. More recently, some researchers used a CoupModel to investigate the interactions between soil *FT* cycles and salinisation as well as other possible causes of salinisation during three winters from 2012 to 2015 in an irrigation district in northern China^5^. Wan et al. (2019) conducted laboratory tests to simulate salt transfer in a real-world cold sodium sulphate soil^6^. Their findings indicated that the maximum salt transport level occurred in the frozen fringe zone of the soil, and the salt expansion had an accumulative effect, which was positively correlated with the number of *FT* cycles. Another study also pointed out that the *FT* processes could lead to more water migrating to interdune than that to sand dunes in the rhizosphere of Horqin Sandy Land, northern China^1^, as supported by Wang et al. (2019) in dam and slope farmland on the Loess Plateau, China^3^. It is worth noting that these aforementioned studies indicated that soil water-salt migrations were closely related to soil *FT* processes and also highlighted the importance of understanding the migrated behaviours of soil water and salt in responses to soil *FT* processes in ensuring effective agricultural managements.

Currently, the western Songnen Plain, located in northeast China, has over 3.4 × 10^6^ ha of saline-sodic soils that cover over 19.0% of its total area, which is considered as one of the three most prominent saline-sodic soil regions worldwide^7^. Ongoing salinisation and alkalisation have resulted in serious degradation of arable land, thereby posing a severe threat to regional agricultural productivity and sustainability^7^. Saline-sodic soils undergo seasonal freezing and thawing because this area is situated in arid and semiarid climatic zones, with freeze events occurring in mid-November and thawing occurring at the end of May in the following year. This regional *FT* period consequently lasts half a year.

However, there are disparities in the literatures regarding the impact of *FT* cycles on soil salinisation in the study area, which are the subject of active debate and require further study. Some researchers suggested that ‘the critical depth of ground water’ controls the ‘eruption’ of salification during the spring, and that the effluence of *FT* cycles on soil salinisation should be ignored^8^. This suggestion seems to contradict the local practices, wherein phreatic water is the only type of water that can affect soil salinisation. Frozen soil layers sever the soil water exchange between the upper soil and groundwater before thawing is complete^9^; therefore, soil salinisation during the spring does not depend on the groundwater level. In contrast, other studies reported that salt along with soil water moved towards the frozen layer and salinity increased in the frozen layer^10,11^. Then, the intensively incremental occurrence of salinity within the topsoil resembled an ‘eruption’ of salinity during the spring thawing period^12,13^. Wang et al. (2009)^14^ reviewed the causes of soil alkalisation in the Songnen Plain and emphasised that winter freezing led to latent salt accumulations that induced further soil salinisation during the subsequent spring. This was consistent with the findings of Zhang and Wang (2001)^12^, who demonstrated that soil salinisation occurred as a strong response to freeze–thaw, yet which has been ignored by some studies^8^. However, these abovementioned studies on soil salinisation related to *FT* processes in the studied region have far been limited to theoretical speculations and qualitative descriptions. Consequently, there is a lack of quantitative experimental investigations into the soil water and salt migration behaviours in response to *FT* processes, spatiotemporal characteristics of soil water and salt and their related conditions. Therefore, the *FT* mechanism behind soil salinisation remains unclear. Furthermore, appropriate land use and management approaches can maintain and decrease the soil salinity and alkalinity, thereby improving the soil quality and health. Minimal information, however, is available on how soil water and salt are migrated during the soil *FT* period in saline-sodic soils of different landscapes.

Therefore, this study aims to (1) determine the characteristics of soil *FT* processes as influenced by different landscapes, (2) explore the influences of *FT* processes on water-salt migration in saline-sodic soil and (3) elucidate the underlying mechanism of soil salinisation-alkalisation during the spring in the western Songnen Plain, China.

## Materials and methods

### Study area

**Figure 1:**
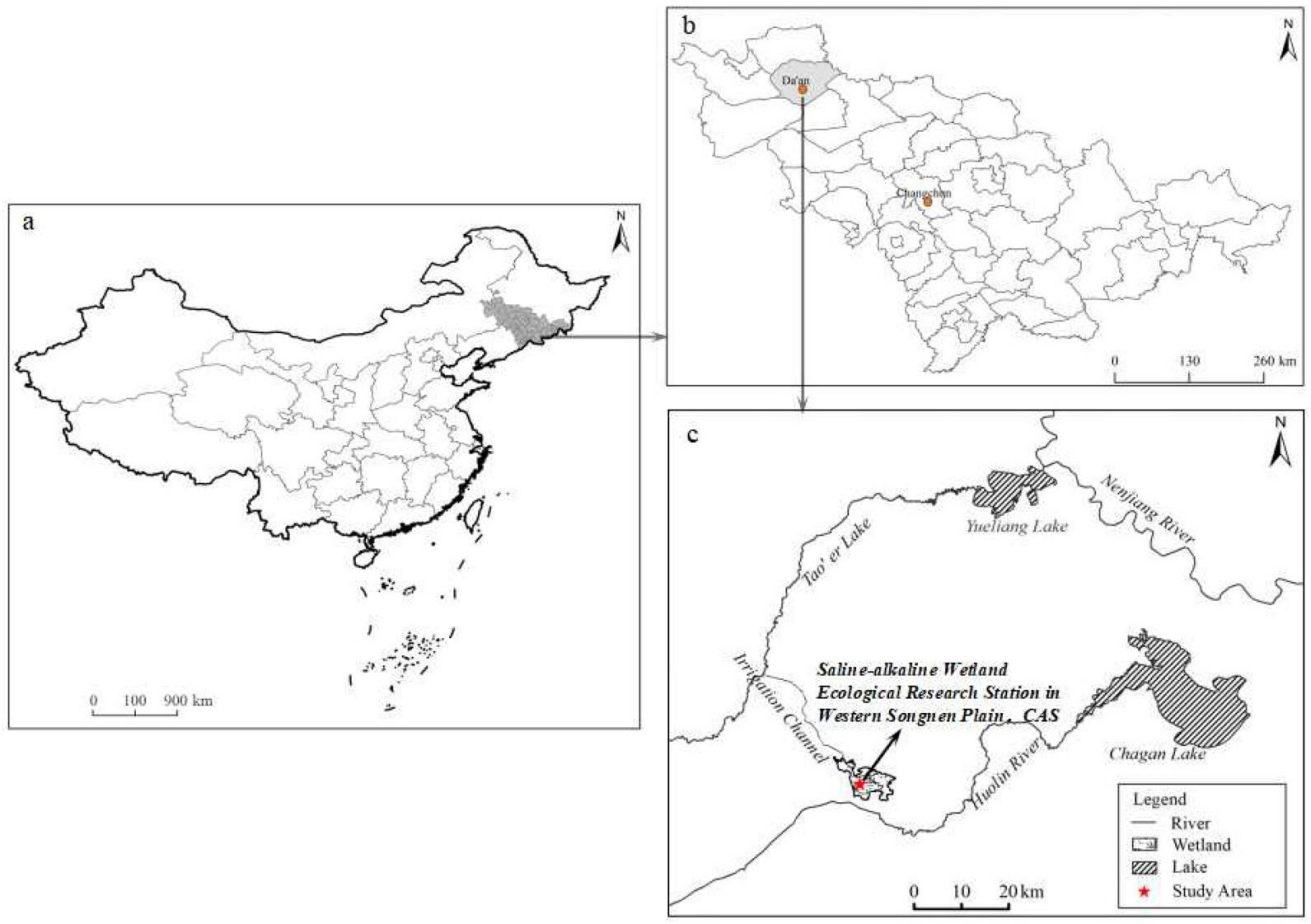
Map of region and study area. Location map of China (a). Location map of Songnen Plain, China (b). Location map of the study area in Western Songnen Plain in Northeast China (c). This map was generated by ArcGIS 10.2 (http://www.arcgis.com/features/index.html).

The study area is located at the Saline-alkaline Wetland Ecological Experiment Station (123° 15′–123° 21′ E, 45° 13′–45° 16′ N) (Fig. 1), in northeastern China’s western Songnen Plain. The region is characterized by a temperate, arid and semiarid continental monsoon climate with an annual average ambient temperature of 4.3 °C, which varies from − 20 °C in January to 26 °C in July. The average annual precipitation is approximately 396 mm, and 70%–80% of the rainfall occurs between June and August. The annual average evaporation is approximately 1817 mm, and the frost-free period is approximately 125–135 days. This area is subjected to seasonal *FT* cycles with freezing beginning at the end of October and thawing occurring in June of the following year. The soils of the study area are alkali-saline, and are classified as Solonetz in the World Reference Base for Soil Resources (IUSS Working Group, 2015). The groundwater depth fluctuates between 1 and 3.2 m.

### Experimental design and soil sampling

**Figure 2:**
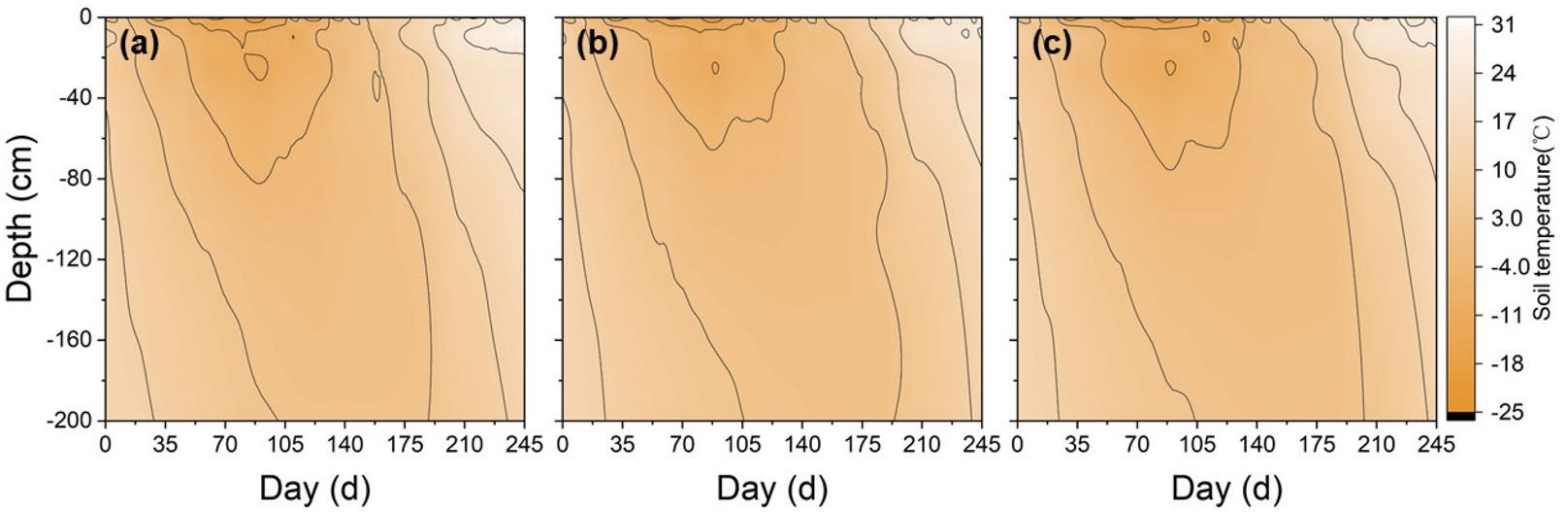
Contour map of soil temperature in farmland (**a**), *LT* grassland (**b**) and *AS* land (**c**) from 24 October, 2015 to 25 June, 2016.

**Figure 3:**
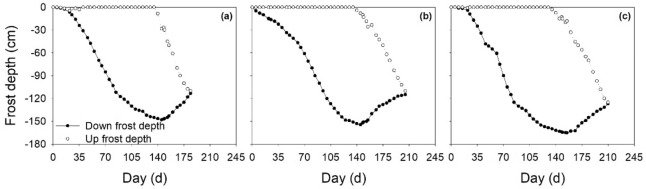
Evolution of soil frost depths in the farmland (**a**), *LT* grassland (**b**) and *AS* land (**c**) from 24 October, 2015 to 25 June, 2016.

The main vegetation in the studied area is natural grassland dominated by *Leymus chinensis* (Trin.) Tzvel (*LT*). Some of this grassland has degraded into alkali-spot land because of the soil salinisation-alkalisation that has resulted from natural and anthropogenic causes, and other grasslands have been reclaimed as maize fields since 2000 to reduce the need for arable land resources^15^. Thereby, three typical plots of *LT* grassland, farmland and *AS* land were selected for field monitoring. The 20 m × 30 m monitoring sites were centrally located in these representative landscape units with a total area of approximately 200 m × 300 m. Three replicates of each monitoring location at intervals of 15 m were selected in each landscape to minimize errors caused by soil heterogeneity. Table 1 lists the soil physical and chemical properties of the selected landscapes. Soil samples were collected from the 0–200 cm soil layers at intervals of 10 cm once every 15 days from October 2015 to June2016. Other samples were air-dried and then separated from root materials before passing through a 2-mm sieve to allow for analysis. In addition, the thickness of snow cover was simultaneously determined when collecting soil samples. The soil temperatures at depths of 5, 20, 40, 70, 100, 150 and 200 cm were monitored via platinum resistance temperature probes (Pt-200, Beijing Qudao, China) with an accuracy of 0.1 °C. Frost tubes (3 cm in diameter) were placed and used to measure frost depths. The soil temperature and frost depths were monitored at 09:00 once in every 5 days (Figs. 2, 3). Ambient temperature was monitored at a weather station and groundwater level data were obtained from local hydrological stations.

**Table 1:** The soil physical and chemical properties of farmland,* LT *grassland and *AS* land at different depths in study area.

Landscapes	Soil depth (cm)	pH	EC (mS/cm)	Salt content (mg/kg)	Organic matter (%)	Clay (%)	Silt (%)	Sand (%)	Bulk density (g/cm^3^)
Farmland	0–30	8.16	0.06	84.82	0.25	2.23	5.75	92.02	1.57
	30–100	8.02	0.08	99.09	0.42	1.58	3.61	94.81	1.63
	100–135	7.87	0.12	128.70	0.28	2.34	5.13	92.53	1.56
	135–150	8.00	0.13	143.18	0.30	5.12	10.41	84.47	1.44
LT grassland	0–25	8.45	0.26	278.50	2.50	3.39	7.69	88.91	1.43
	25–60	8.38	0.66	495.00	0.52	6.53	14.88	78.59	1.53
	60–90	8.01	0.68	632.65	0.34	7.25	20.29	72.46	1.54
	90–150	7.98	0.49	585.88	0.28	5.92	10.28	83.8	1.59
AS land	0–20	11.25	1.68	2218.50	0.16	4.90	8.47	86.62	1.73
	20–55	10.62	0.91	972.33	0.26	4.44	7.96	87.59	1.55
	55–90	9.52	0.82	770.00	0.24	6.79	9.86	83.35	1.57
	90–150	9.61	0.69	627.19	0.19	7.30	14.95	77.74	1.61

### Soil analysis

The 1:5 soil to water extracts were used for the analyses of soluble Na^+^, Mg^2+^, K^+^, Ca^2+^, CO_3_^2−^, HCO_3_^−^, Cl^−^, SO_4_^2−^, EC and pH values. The total soluble salt content was calculated by summing the contents of soluble Na^+^, K^+^, Ca^2+^, Mg^2+^, CO_3_^2−^, HCO_3_^−^, Cl^−^and SO_4_^2-^^16^. The soil organic content, the cation exchange capacity (*CEC*) and exchangeable Na^+^ were determined by the dichromate oxidation method, sodium acetate and CaCO_3_–CO_2_ exchange neutral titration method, respectively^16^. The soil texture was determined using a laser diffraction particle size analyser (MS-2000, Malvern Instruments Ltd, UK).

The sodium adsorption ratio (*SAR*) ((mg/L)^1/2^) was calculated as follows:

1 $$SAR = \frac{{\left[ {{\text{Na}}^{ + } } \right]}}{{\sqrt {0.5\left[ {{\text{Ca}}^{2 + } } \right] + 0.5\left[ {{\text{Mg}}^{2 + } } \right]} }},$$

where [Na^+^], [Ca^2+^] and [Mg^2+^] are cation concentrations (mg/L).

The exchangeable sodium percentage (*ESP*) was calculated using the ratio of the exchangeable [*Na*^+^] to the *CEC* defined as follows:

2 $$ESP(\% ) = \frac{{\left[ {Na_{{\text{s}}} } \right] \times 100}}{CEC},$$

where [*Na*_s_] is the content of exchangeable sodium (cmol/kg), and *CEC* is the cation exchangeable capacity (cmol/kg).

The water storage change rate (*WSCR*) (cm/d) of different soil layers was calculated as follows:

3 $$WSCR = \frac{{\left( {WC_{t + \Delta t} - WC_{t} } \right) \times \Delta z}}{\Delta t},$$

where $\Delta t$ (*d*) is the time interval between two measurements of *SWC* (cm^3^/cm^3^), and $\Delta z$ (cm) is the difference in distance between two depths.

The freeze–thaw rate (*FTR*) (cm/d) was calculated using Eq. (4):

4 $$FTR = \frac{{FD_{t + \Delta t} - FD_{t} }}{\Delta t},$$

where Δ*t* (d) is the time interval between two measurements of the soil frost depth (*FD*) (cm).

### Statistical analysis

The statistical analyses were performed using the SPSS 19.0 statistic software package (SPSS, Inc., Chicago, IL, USA). Pearson correlation analyses were applied to determine the correlation between soil salinity, alkalinity, water, soil temperature, groundwater table and frost depth. Significant differences were evaluated at the 0.05 levels.

## Results

### Soil freeze–thaw characteristics

**Figure 4:**
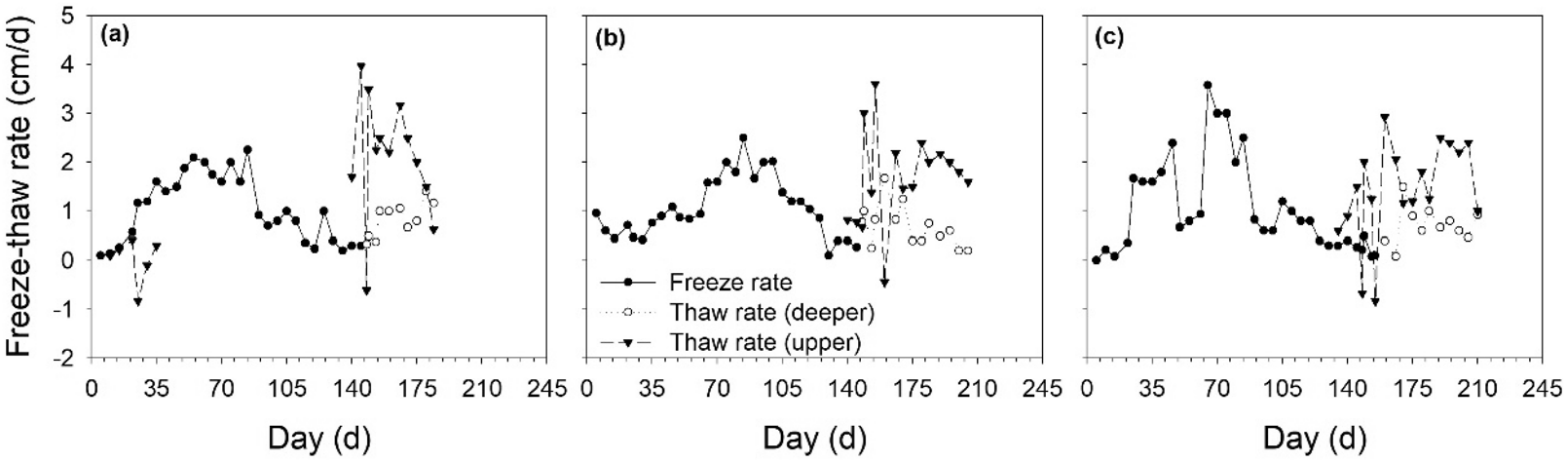
Soil freeze–thaw rate of farmland (**a**), *LT* grassland (**b**) and *AS* land (**c**).

Figures 2 and 3 show the soil *FT* processes versus soil depth associated with soil temperature. As the ambient temperature decreased below 0 °C in late October 2015, soil temperatures decreased accordingly, and then the soil began to freeze from the topsoil down into the deep layers and entered a frozen state. Until the temperature increased to above 0 °C in mid-March of 2016, the soil temperature also rose, and the surface soil began to thaw; However, the deeper soil layer remained frozen. When the temperature of the subsoil also rose above 0 °C, the upper and lower layers of the frozen soil begin to thaw, both downwards and upwards until the soil thawed completely in June 2016. The freezing rate was the largest for the *AS* land, followed by the LS land, and then the farmland (Fig. 4). The maximum freezing rates of the farmland, *LT* grassland and *AS* land were 2.25, 2.50 and 3.58 cm/d, respectively. On 17 March, 2016, the maximum frozen depth occurred in the *LT* grassland, whereas this maximum occurred in the farmland and *AS* land at a slightly later date (27 March, 2016). Accordingly, the soil freeze durations were 184, 205 and 220 days with the maximum frost depth at 148, 155, and 165 cm, respectively, for the farmland, *LT* grassland and *AS* land (Fig. 3). The farmland, *LT* grassland and *AS* land all thawed bi-directionally until late-April (at 110–115 cm depth), mid-May (at 110–113 cm depth) and late-May (at 120–125 cm depth), respectively. The date of thawed bi-directionally on farmland was about 21 days earlier than that for the *LT* grassland and *AS* lands.

### Profile distributions of the SWC

**Figure 5:**
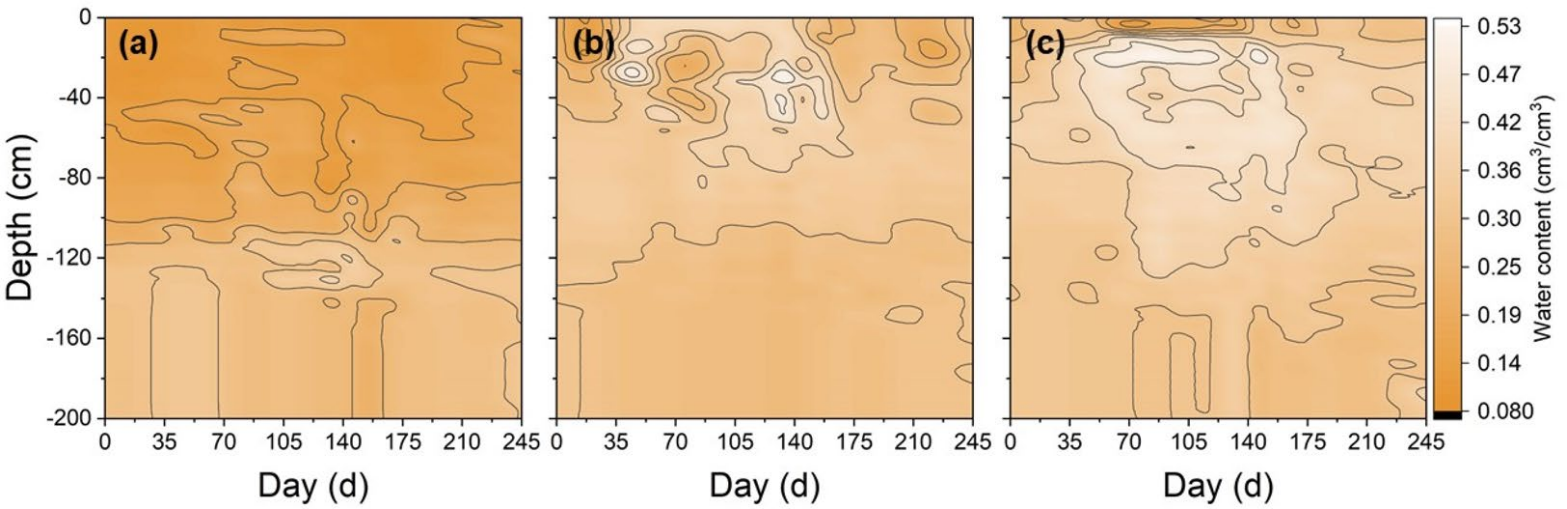
Contour map of soil water of farmland (**a**), *LT* grassland (**b**) and *AS* land (**c**) from 24 October, 2015 to 25 June, 2016.

In the seasonally frozen soil region, soil *FT* processes were accompanied by water and heat transfers. Figure 5 shows the contour map of the *SWC* under *FT* conditions for all the three landscapes. The *SWC* (0–200 cm) in the *LT* grassland and *AS* land increased by 9.70% and 17.43% during freezing, respectively, and decreased by 4.03% and 11.46% during thawing, respectively. Conversely, the *SWC* in farmland decreased by 1.59% during freezing and increased by 5.63% during thawing. The average S*WC* (0–200 cm) of the farmland, *LT* grassland and *AS* land increased by 1.15%, 1.93% and 3.56%, respectively, during the *FT* period. Furthermore, *SWC* varied with the soil profiles in the different landscapes (Table 2). In *LT* grassland, the *SWC* of 0–80 cm increased and the *SWC* of 80–200 cm decreased during the freezing period, while the *SWC* of 0–100 cm increased and the *SWC* of 100–200 cm decreased during the thawing period. On the contrary, The *SWC* of *AS* land decreased at 0–5 cm depth and increased at 5–200 cm depth in freezing process, while it increased at 0–5 cm depth and decreased at 5–200 cm depth in thawing process. Compared with *LT* grassland and *AS* land, water migration and changes in different soil layers of farmland were more complicated. During the freezing period, the *SWC* of 0–50 and 90–130 cm in farmland increased (7.81% and 14.56%), and the *SWC* of 50–90 and 130–200 cm decreased (-14.99% and -11.76%). In the thawing period, the *SWC* of 0–50 and 100–130 cm of farmland decreased (-14.64% and -11.94%), and the *SWC* of 50–100 and 130–200 cm increased (29.25% and 9.36%). In addition, the *SWC* change rate of the upper soil layer was greater than that of the deeper soil layer (Table 2).

**Table 2:** Soil water increase ratio of farmland, LT grassland and AS land in freezing period and thawing period.

Landscapes	Soil depth (cm)	Water increase ratio in freezing period (%)	Soil depth (cm)	Water increase ratio in thawing period (%)
Farmland	0–50	7.81	0–50	− 14.64
	50–90	− 14.99	50–100	29.25
	90–130	14.56	100–130	− 11.94
	130–200	− 11.76	130–200	9.36
LT grassland	0–80	45.59	0–100	− 11.94
	80–200	− 14.22	100–200	9.36
AS land	0–5	− 3.58	0–5	27.59
	5–200	17.96	5–200	− 12.46

**Figure 6:**
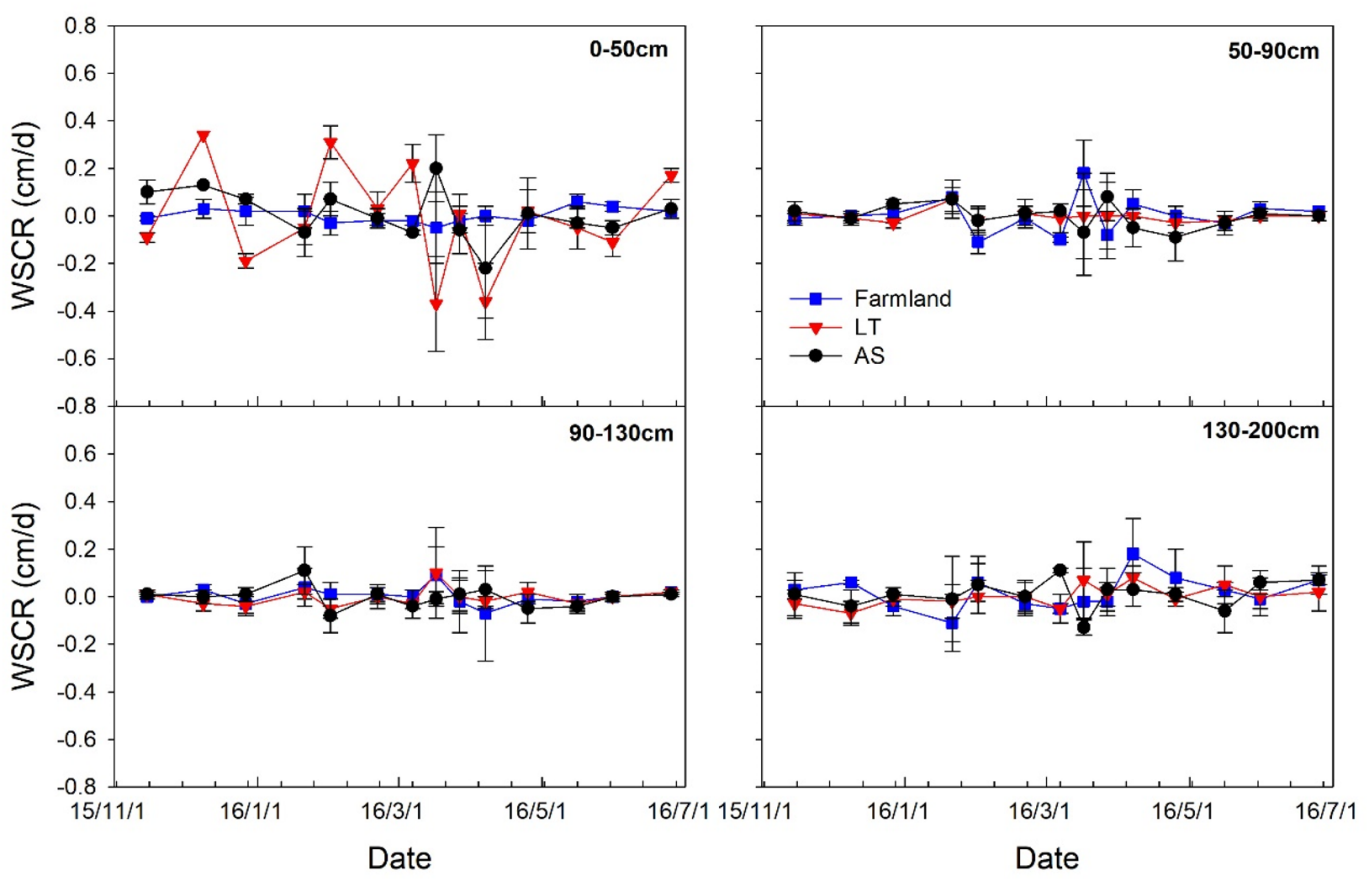
Dynamics of soil water storage change rate (*WSCR*) of four soil layers in farmland, *LT* grassland and *AS* land.

Soil water migrations were clearly observed in the soil profiles of the different landscapes (Fig. 6). The *WSCR* value was higher in the 0–50 cm soil layer than those in other layers (Fig. 6). The largest fluctuations occurred in the *LT* grassland, whereas the smallest occurred in the farmland, thus indicating that the highest and lowest amounts of water migrated in the former and latter locations, respectively, during the *FT* processes. There were also variations in the *WSCR* at a depth of 110–200 cm for all the three landscapes, which indicated that soil water obviously migrated at these soil layers.

### Profile distributions of the soil salinity

**Figure 7:**
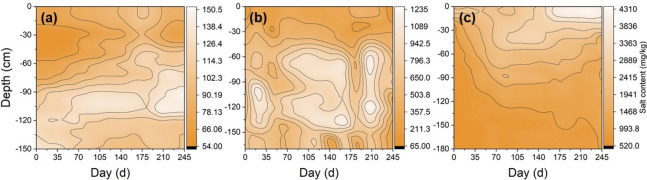
Contour map of soil salt content in farmland (**a**), *LT* grassland (**b**) and *AS* land (**c**) from 24 October, 2015 to 25 June, 2016.

A contour map of the soil salt profiles is plotted in Fig. 7. Remarkably, the soil salt moved upward overall, and the profile distribution of the soil salt expanded during the soil *FT* processes (Fig. 7). In the farmland, the salification layer moved upwards and expanded from the 130–150 cm depth before freezing to 80–110 cm after completely thawing at the end of April 2016. In the *LT* grassland, the soil salification layer rose upwards and expanded from the 70–100 cm depth before freezing to the 60–130 cm depth after completely thawing in mid-May 2016. In the *AS* land, different from farmland and *LT* grassland, soil salt tended to accumulate on the soil surface with a significantly higher salinised ratio (*P* < 0.05) than those at other soil layers (Table 3) after fully thawing at the end of May 2016, whereby the salification layer moved upwards to 10–20 cm from 30–40 cm. The salt content of the salification layers of the farmland, *LT* grassland and *AS* land reached 125.20, 1,088.35 and 4,209.51 mg/kg, respectively, and increased by 31.27%, 65.25% and 79.82%, respectively, at the end of thawing.

**Table 3:** Soil salinized ratio of farmland, LT grassland and AS land in freezing period and thawing period.

Landscapes	Soil depth (cm)	Salt at the beginning of freezing (mg/kg)	Salt at the end of freezing (mg/kg)	Salt at the end of thawing (mg/kg)	Salinized ratio in freezing (%)	Salinized ratio in thawing (%)
Farmland	0–10	74.10	87.80	102.49	18.49 a	16.73 a
	10–100	74.89	99.34	111.46	33.80 a	14.81 a
	100–150	126.29	109.82	110.27	− 12.27 b	0.68 b
LT grassland	0–10	114.63	273.56	412.53	138.65 a	50.80 a
	10–30	315.44	377.09	255.73	22.01 b	− 32.60 b
	30–130	555.65	908.80	1009.07	63.28 b	10.88 ab
	130–170	635.52	824.05	740.81	29.46 b	− 10.21 b
AS land	0–10	2322.67	2765.84	4209.50	19.08 b	52.20 a
	10–40	965.78	2990.47	2894.90	213.71 a	− 2.84 ab
	40–110	780.33	1862.35	1456.57	136.37 ab	− 20.13 b
	110–180	557.74	910.51	955.95	62.71 b	5.47 ab

Letters represent multiple comparison results and the dissimilar letter indicate a significant difference (*P* < 0.05).

As shown in Table 3, a freeze-induced salt enrichment clearly occurred throughout the whole frozen layer with one exception for the 100–150 cm layer in the farmland. The freeze-induced salinised ratios changed by approximately − 12.27% to 33.80%, 22.01%–138.65% and 19.08%–213.71% during the freezing period in farmland, *LT* grassland and *AS* land, respectively. During the freezing period, the salinised ratios were significantly higher (*P* < 0.05) in the upper soil than those in the deeper soil. Thus, soil salt accumulated in the frozen layer mainly came from the deeper soil profiles. In contrast to those observed before freezing, at the end of thawing period, the surface soil salt contents had increases of 16.73%, 50.80% and 52.20% in the farmland, *LT* grassland and *AS* land, respectively, except that in the 10–30 cm deep layer in *LT* grassland, which reduced by 32.6% (Table 3). Furthermore, the salinised ratio in the surface soil was significantly higher (*P* < 0.05) than that in the subsurface soil, and the subsurface soil was desalinised (Table 3). This phenomenon was most pronounced in *AS* land, followed by *LT* grassland, and then farmland.

### Profile distributions of the soil alkalinity

**Figure 8:**
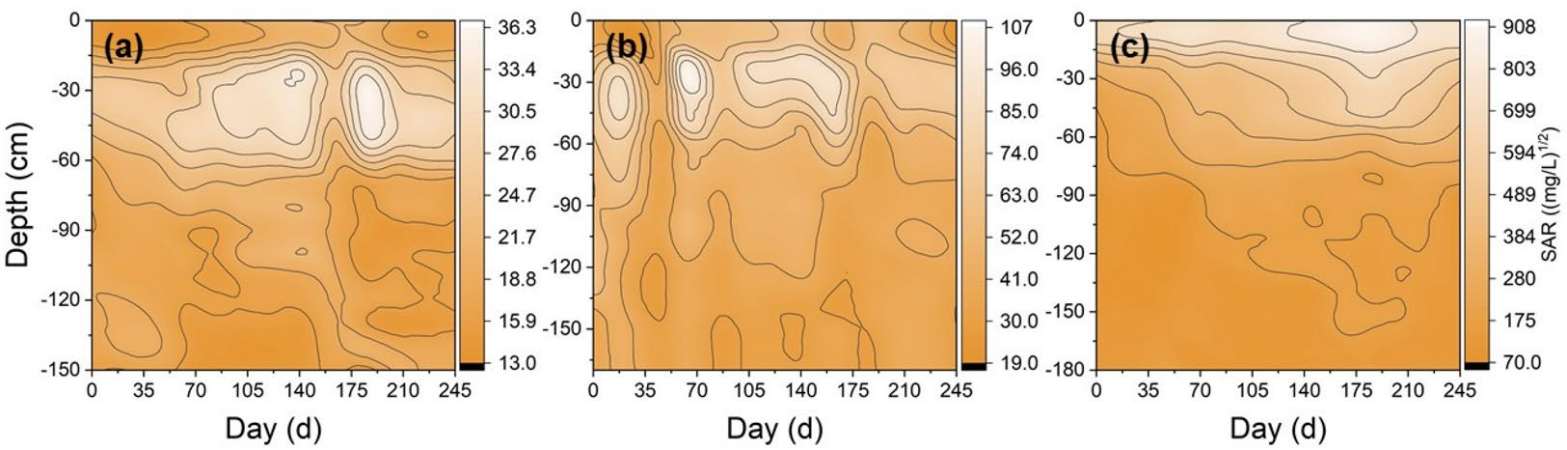
Contour map of soil SAR in farmland (**a**), LT grassland (**b**) and AS land (**c**) from 24 October, 2015 to 25 June, 2016.

Figure 8 shows the changes in the *SAR* profile distributions during the *FT* processes. These *FT* processes resulted in a significant increase in the *SAR* of the upper soil in all three landscapes (Fig. 8), thereby resulting in soil alkalisation (Table 4). The *SAR* increase ratios substantially varied with land types and soil depths undergoing the *FT* processes (Table 4). After the soil completely thawed, *SAR* increased by 41.81%, 28.83% and 78.70% at depths of 0–50 cm in the farmland, 0–40 cm in the *LT* grassland and 0–70 cm in the *AS* land, respectively (Table 4). Furthermore, *SAR* increase ratios for the *AS* land at the 0–70 cm depth were 46.87% and 63.37% higher, respectively, than those in the *LT* grassland and farmland, respectively.

**Table 4:** Soil SAR increase ratio of farmland, LT grassland and AS land in freeze–thaw processes.

Land types	Soil depth (cm)	SAR at the beginning of freezing ((mg/L)^1/2^)	SAR at the end of thawing ((mg/L)^1/2^)	SAR increase ratio (%)
Farmland	0–10	15.58	21.84	40.18
	10–50	23.54	33.77	43.43
	50–100	16.84	17.35	3.05
	100–150	17.89	16.82	− 5.99
LT grassland	0–10	36.64	54.48	48.68
	10–40	61.96	67.52	8.97
	40–170	50.91	40.67	− 20.10
AS land	0–10	631.86	778.33	23.18
	10–70	216.99	508.25	134.22
	70–130	119.83	192.03	60.25
	130–180	122.79	133.09	8.38

The *FT* also induced *ESP* increased substantially over time in the 0–60 cm deep layer, and increased by 86.58%, 102.50% and 153.08% for the farmland, *LT* grassland and *AS* land, respectively (Table 5), demonstrating that the influences of the *FT* processes on the soil alkalisation in the *AS* land were greater than those in the farmland and *LT* grassland in the study area.

**Table 5:** Soil ESP increase ratio of farmland, LT grassland and AS land in freeze–thaw processes.

Land types	Soil depth (cm)	ESP at the beginning of freezing (%)	ESP at the end of thawing (%)	ESP increase ratio (%)
Farmland	0–20	5.92	10.76	81.76
	20–60	7.73	14.80	91.40
	60–100	10.84	11.85	9.34
	100–150	12.50	12.39	− 0.92
LT grassland	0–20	15.88	33.33	109.89
	20–60	18.91	36.89	95.12
	60–100	25.22	29.38	16.48
	100–150	25.25	20.57	− 18.54
AS land	0–20	74.80	184.65	146.87
	20–60	46.80	121.35	159.29
	60–100	45.51	53.35	17.23
	100–150	40.48	42.22	4.28

### Relationships between soil salinity, soil alkalinity, soil water, soil temperature, groundwater table and frost depth

The relationships between soil salinity, alkalinity, *SWC*, soil temperature, groundwater table and frost depth are presented in Table 6. It can be seen that there are significant positive correlations between the soil salinity and frost depth, as well as *SWC* (*P* < 0.01) within the three landscapes. The correlation between the soil salinity and soil temperature was negatively correlated. A significant negative correlation also occurred between soil salinity and the groundwater table (*P* < 0.01). Furthermore, a significant negative correlation was also found between the soil frost depth and soil temperature (*P* < 0.01).

**Table 6:** Correlations between soil salinity, alkalinity, soil water, soil temperature, groundwater table and frost depth.

	Salt content	SAR	Water content	Soil temperature	Groundwater table	Frost depth
Salt content	1	.910**	.655**	− .034	− .119**	.194**
SAR		1	.490**	− .028	− .076	.123**
Water content			1	− .029	− .086*	.151**
Soil temperature				1	.401**	− .550**
Groundwater table					1	− .699**
Frost depth						1

**Correlation is significant at the 0.01 level (2-tailed).

*Correlation is significant at the 0.05 level (2-tailed).

## Discussion

### Soil freeze–thaw characteristics in different landscapes

In this study, the *FT* characteristics exhibited certain differences among the three landscapes. *AS* land had the largest frost depth and the longest freeze duration, followed by *LT* grassland, and then farmland (Fig. 3). These differences may be attributed to the differences in dependent soil physical properties, soil surface covers and initial soil water contents of the landscape^17,18^. The denser soil structure of the *AS* land quickened the more spread of the cold from the upper soils to the lower soils than *LT* land and farmland during freezing. Furthermore, *AS* land had the lowest snow cover and no residue, which promotes heat transport at the soil–atmosphere interface^19^. Therefore, the soil temperature decreased rapidly with the air temperature, resulting in a significant increase in the frost depth and freezing rate of *AS* land. These was in accordance with the conclusions of Iwata et al. (2010)^20^, who clearly demonstrated that the reduction in a snow cover deposition could cause a dramatic increase in the frost depth, as well as those of Fu et al. (2018)^2^ who reported that the decrease in snow cover strengthened the actions of soil temperature on freezing front. In addition, the higher *SWC* in the *LT* grassland (0.21 cm^3^/cm^3^) and *AS* land (0.32 cm^3^/cm^3^) would slow down soil temperature changes^21^, because more heat release from the soil when soil freezes, or more heat is needed when soil thaws. Consequently, the wetter conditions in the *LT* grassland and *AS* land would postpone the freeze–thaw processes, as indicated by other researches^17^. Similar results were obtained in the study of Yi et al. (2014) on soil freeze–thaw characteristics of different landscapes in the Heihe River Basin, Gansu, China^22^.

### Influence of freeze–thaw process on the soil water content (SWC)

In this study, the freezing process led to an upward enrichment in soil water within different landscapes in the study area (Fig. 5). One possible reason for this phenomenon was that the soil temperature gradient drove the upward flux of water towards the frozen layers, and water finally accumulated in the frozen layers^23,24^. However, during the spring thawing, soil water in the upper soil layer and the deeper soil layers decreased and increased, respectively (Table 2). This was because soils thawed bi–directionally, the water above the frozen layer moved upwards and ultimately intensively evaporated away, whereas the water below the frozen infiltrated into the deeper layers. These results agreed with the findings of Zhang and Wang (2001)^12^, Wang et al. (2009)^14^ and Bing et al. (2015)^4^. Furthermore, this freeze-induced soil water enrichment in the frozen zone can facilitate to soil water conservation by reducing evaporation and seepage, thus maintaining a high water content^1,3,22^, which can be helpful to farming and plant germination in the following spring. However, in this study there were obvious differences in profiled water redistributions upon freezing in different landscapes. The *SWC* in the *AS* land at a depth of 0–5 cm decreased during freezing and increased during thawing. This may be attributed to fierce regional winds, no plant residue on the surface, lack of snow cover and frequent heat exchanges between the surface soil and air during winter in the study area. Furthermore, because of the higher initial moisture content, the greater frost depth and intensity in the *AS* land, the water in the frozen layer continuously replenished the surface soil, and even produced internal runoff during spring thawing, despite an intensification of evaporation. This occurrence thereby increased the surface *SWC*, which proved the conclusions of Iwata et al. (2010)^20^, Nagare et al. (2012)^25^ and Wu et al. (2019)^21^. In addition, the profiled soil water migration rates in the farmland, *LT* grassland and *AS* land were substantially different during the *FT* processes. It was the highest in the *LT* grassland, whereas the lowest in the farmland. This is because the *LT* grassland was less salinised than the *AS* land and surface soil of the former had the highest organic matter content (0–25 cm, 2.50%) (Table 1), which resulted in a good soil structure that facilitated a better movement of soil water compared to the *AS* land. Therefore, more water migrated in the *LT* grassland than that in *AS* land during the *FT* processes. However, for the farmland, the lower initial moisture content (0.11 cm^3^/cm^3^) and soil compaction caused by farming activity over many years inhibited soil water migration. Furthermore, the *FT* affect soil physical properties, such as soil structure, soil cracking, soil thermal properties and heat flux, which were also an important reason explaining the difference of water migration in soil profiles of different landscapes. For example, frozen soils are divided into layered and reticulate structures by ice, resulting in a higher soil water permeability coefficient; thus, water can be quickly discharged from cracking during soil thawing^23,24,26^. Additionally, the groundwater table declined during freezing and rose during thawing, thus suggesting that a mutual transport occurred between the soil water and groundwater in deeper soil.

### Influence of freeze-thaw process on the soil salinity and alkalinity

According to the data obtained from this study, the profiled soil salinity distributions were characterised by an accumulation of soil salt towards the frozen layer with soil water during the freezing. Consequently, the salt content obviously increased throughout the entire frozen layer, which experimentally verified the findings of Stahli and Stadler (1997)^27^ and Wang et al. (2009)^14^. A possible explanation for these results was that the soil salt along with water in the deeper unfrozen layer and groundwater both moved upward towards the frozen layer because of temperature gradient between the frozen and unfrozen layer. In fact, the freeze-induced soil salt migration was exceedingly complex and could not be solely attributed to the temperature gradient. Instead, this dynamic represented the integrated result of many factors, such as land use, initial soil water, soil salinisation, soil temperature, groundwater level. Furthermore, our results also showed that the salinised ratio in the upper soil profile was substantially higher than that in the deeper soil profile during freezing. This behavior may be attributed to the liquid water occurring in the frost layer and temperature gradient forcing the liquid water to carry the salt upwards. Some researchers have observed that it is possible for liquid water to exist as membrane water, wherein its thickness gradually becomes thinner from the deep soil to the upper soil, thereby causing salt to move upwards along with water^28^.

Furthermore, our study showed that the salification layer moved upwards and expanded, and the surface soil exhibited the significant salt accumulations in the *LT* grassland and *AS* land during spring thawing. This appears to experimentally explain the phenomenon of topsoil salt explosive increases that resemble an ‘eruption’ during spring thawing^12,14^. The results were in accordance with Han et al. (2010)^29^, who pointed out that the surface soil salinity increased rapidly in spring because of strong evaporation, more *FT* cycles and longer freezing durations. This is because the quantity of evaporation is five times higher than the amount of rainfall in the western Songnen Plain; thus, this intense soil evaporation induces a redistribution of the accumulated salt in the frozen layer, and transports a large quantity of salt upwards to the surface. More importantly, these findings revealed that *FT* processes were mainly responsible for the obvious soil salinisation in our study, which aligns with the analysis of Bing et al. (2015)^4^, who determined that *FT* processes are the main driving force of soil water and salt movement and are responsible for soil salinisation during the spring in cold and arid regions. However, these results slightly contradicted the findings of Wang. (1993)^8^, who noted that the surface soil salt ‘eruption’ in spring was controlled by ‘the critical depth of ground water’ rather than *FT* actions, yet which contradicted the local practical condition of using phreatic water as the only water source influencing the soil salinisation in this study area. The water exchanges were blocked by the frozen layers between the soil surface and underground water; therefore, soil salinisation during the spring was not related to groundwater^5,12^. However, their findings slightly contradicted with the results observed in our study that suggested that bi-directional thawing also possibly caused the salt under the freezing layer to accumulate in the middle soil profile. This was because the thawed water carrying salt infiltrated towards the deeper soil into the groundwater, which implied that the profiled salt distributions had a relationship with the groundwater. Moreover, the results from our study also revealed that landscapes affected the salification of the soil surface and the desalinisation of the subsurface soil, with the trend being *AS* land > *LT* grassland > farmland. This discrepancy may be interpreted by four aspects. Firstly, the initial soil salt content of *AS* land was 19.3 times higher than that of the *LT* grassland, consequently causing a higher accumulation ratio of soil salt, as indicated by Wan et al. (2019)^6^, who observed that the salt crystallisation increased the salt migration during the freezing process, and that salt migration was positively correlated with the salt content. Secondly, *LT* grassland had a larger coverage area and a higher litter amount reduced the quantity of ground evaporation and avoided surface salt accumulation. Thirdly, the improved soil structure of the *LT* grassland, with its larger root system and higher organic content was beneficial to increase infiltration and promote the downward movement of salt from the upper soil layers^30^. Finally, the soil of *AS* land started to thaw earliest because of its lowest freezing point resulted from its highest salt content at corresponding depths, which accelerated the consumption of soil water by evaporation. Additionally, the salinised ratio of the farmland was weaker than that of *AS* land and *LT* grassland, which was attributed to its lower initial salt content (64.73 mg/kg), initial water content (0.11 cm^3^/cm^3^), lower frost depth and intensity in farmland^21,25^.

The soil *SAR* and *ESP* have been recommended the sensitive indicators of soil alkalisation for a soil alkalisation assessment in the Songnen grassland^31^. In this study, *FT* cycles induced the increases in the *SAR* and *ESP* in the upper soil layers for all three landscapes (Fig. 8 and Table 5), which implied that the *FT* processes not only contributed to soil salinisation but also to soil alkalisation. As indicated in Table 6, the soil salinisation within the frozen soil layer shows a significantly positive correlation with the soil alkalisation, which was similar to the findings observed by Yu et al. (2018)^31^. This occurrence may be mainly attributed to the fact that the salts migrating towards the frozen layer had a prevalence of NaHCO_3_ and Na_2_CO_3_^13^. Wang et al. (2009)^14^ also reported that soil *FT* were one of the most important causes of soil salinisation and alkalisation in the western Songnen Plain and further proved that the influence of groundwater could not be ignored. Groundwater in the study area comprises a weak mineralised water of NaHCO_3_ type, where Na^+^, CO_3_^2−^ and HCO_3_^−^contents can be up to 853.55 mg/L, the salinity is as high as 1.21 g/L and the *SAR* can reach 88.65. Accordingly, groundwater migrating upwards due to soil freezing induced soil both salinisation and alkalisation, which accelerated soil degradation^13^. Conversely, some studies have reported that *FT* cycles had no significant effects on soil *CEC* or exchangeable Ca^2+^ and Mg^2+^ but significantly decreased the exchangeable K^+^^32^ that indicated that *FT* cycles can possibly reduce the soil alkalisation, which was different from our results. The cause of this difference is not clear that is the integrated result of various factors, such as soil types, vegetation types, microbial activity, ground level, and so on. The experimental conditions in this study were different from Hinman (1970)^32^ in which soils were fumigated and sterilize without groundwater exchange and vegetation. Furthermore, the influences of soil *FT* on soil alkalisation varied with soil types and soil depths. In this study, the *FT*-induced soil alkalisation in the *AS* land was more pronounced than that in the farmland and *LT* grassland (Table 4). This may be a comprehensive consequence of land use, groundwater levels, topography, soil-human activities, and so on.

### Hypothetical mechanism of freeze–thaw influences on soil salinity and alkalinity

The *FT* process caused variations in profiled soil water and salt distributions^12^, yet the internal mechanism still stayed at an exploration stage. During freezing, the potential head gradients between the frozen and unfrozen zones created by the temperature gradient exerted a certain driving force behind an upward flux of water towards the upper zones^3,25^. Salt, using water as the carrier, also rose towards the upper layer and was finally enriched in the frozen layer, which thereby increased the salinity. The enriched salts in the frozen layer were driven by intense surface evaporation to move towards soil surface and then accumulated, which been characterized as ‘eruptions’ during the spring. Therefore, the intensity of freezing during the winter and the strength of surface evaporation during the spring determined the extent of surface soil salinity-alkalinity.

Moreover, there was sufficient evidence to prove that soil salt migration was related to land use and vegetation. Soil colloidal particles were dispersed most widely in the *AS* land because of the highest Na^+^ contents, and most dispersed fine clay particles moved downward through the subsoil to act as a dense water barrier. Additionally, the poor soil structure of the *AS* land directly slowed down the soil water and salt migration rate on the unfrozen layer and the upward migration of groundwater toward the frozen layer. The relatively superior soil structure in the *LT* grassland promoted soil water and salt removal. Furthermore, various types of vegetation have differentially improved the soil physical, chemical and biological properties^31,33^, and these differential reactions may contribute to the response to *FT* actions. The vegetation coverages and the sizes of their root networks influenced evapotranspiration and soil water percolation, which consequently further influenced the upward water and salt migrations during *FT*. Maize vegetation has been found to have a greater impact than grass vegetation on repairing saline-sodic soils in the study area, and were both found to have superior soil physical properties compared to non-vegetated *AS* land^28^. Therefore, the *FT* processes, as associated with different landscapes and vegetation coverage, controlled soil water and salt migration during the winter and spring, which were mainly responsible for the variations in the soil salinity and alkalinity in the study area.

## Conclusions

In conclusion, the highest frost depth and longest freezing duration occurred in the alkali-spot land, followed by the *Leymus chinensis* (Trin.) Tzvel grassland, and then farmland. Deeper soil water and groundwater moved towards the frozen layer, therefore resulting in the migration and redistribution of soil water and salt during freezing under the temperature gradient and hydraulic gradient. The key finding of this study demonstrates that freeze–thaw processes led to the soil salification layer rising and expanding for all three landscapes. The explosive surface accumulation of salts during the spring thawing originated from the enriched salt in the frozen layers. Therefore, the freeze–thaw processes contributed to the surface soil salinisation and alkalisation in spring. Furthermore, the freeze–thaw-induced soil salt migration was related to land use, whereby that the salt upward migrating intensities exhibited an order of alkali-spot land > *Leymus chinensis* (Trin.) Tzvel grassland > farmland.

## References

[1] Ala M, Liu Y, Wang AZ, Niu CY (2016). Characteristics of soil freeze–thaw cycles and their effects on water enrichment in the. Geoderma.

[2] Fu Q (2018). Effects of soil water and heat relationship under various snowcover during freezing–thawing periods in Songnen Plain, China. Sci. Rep UK.

[3] Wang T (2019). The effects of freeze–thaw process on soil water migration in dam and slope farmland on the Loess Plateau, China. Sci. Total Environ..

[4] Bing H, He P, Zhang Y (2015). Cyclic freeze–thaw as a mechanism for water and salt migration in soil. Environ. Earth Sci..

[5] Wu MS (2019). Simulation of dynamical interactions between soil freezing/thawing and salinisation for improving water management in cold/arid agricultural region. Geoderma.

[6] Wan XS, Gong FM, Qu MF, Qiu EX, Zhong CM (2019). Experimental study of the salt transfer in a cold sodium sulfate soil. KSCE J. Civ. Eng..

[7] Chi CM, Wang ZC (2013). Interrelationships between grassland plant community distribution and soil physical and chemical properties in soda–saline soil regions of Songnen Plain, Northeast China. Chin. J. Ecol..

[8] Wang ZQ (1993). Chinese Salted Soil.

[9] Qiu SW, Zhang B, Wang ZC (2005). Analyses on current situation, causes of formation, and way of management of desertifification in western Northeast Plain of China. Quat. Sci..

[10] Xu, W., Mao, X. S., Tang, K. & Tang, X. L. Study on water and salt migration of saline soil under the condition of freeze–thaw cycles. *Proceedings of the 2019 World Transport Conference (Part 1).* **12**, (2019).

[11] Wu DY, Zhou XY, Jiang XY (2018). Water and salt migration with phase change in saline soil during freezing and thawing processes. Groundwater..

[12] Zhang DF, Wang SJ (2001). Mechanism of freeze–thaw action in the process of soil salinisation in northeast China. Environ. Geol..

[13] Luo JM (2011). Mechanism of soil sodification at the local scale in Songnen Plain, Northeast China, as affected by shallow groundwater table. Arid. Land Res. Manag..

[14] Wang L, Seki K, Miyazaki T, Ishihama Y (2009). The causes of soil alkalinisation in the Songnen Plain of Northeast China. Paddy Water Environ..

[15] Liu Q, Cui BS, Yang ZF (2009). Dynamics of the soil water and solute in the sodic saline soil in the Songnen Plain, China. Environ. Earth Sci..

[16] Bao, S. D. Soil analysis for agronomy, 3rd edn. China Agriculture Press, Beijing, PP 152–196 (in Chinese) 3rd edn. Wiley, New York, pp 230–257 (2000).

[17] Zhao Y, Huang MB, Horton R, Liu F, Peth S, Horn R (2013). Influence of winter grazing on water and heat flow in seasonally frozen soil of Inner Mongolia. Vadose Zone J..

[18] Iwata Y, Hayashi M, Hirota T (2008). Effects of snow cover on soil heat flux and freeze–thaw processes. J. Agric. Meteorol..

[19] Gan L, Peng X, Peth S, Horn R (2012). Effects of grazing intensity on soil thermal properties and heat flux under *Leymus chinensis* and *Stipa grandis* vegetation in Inner Mongolia, China. Soil Till. Res..

[20] Iwata Y, Hayashi M, Suzuki S, Hirota T, Hasegawa S (2010). Effects of snow cover on soil freezing, water movement, and snowmelt infiltration: A paired plot experiment. Water Resour. Res..

[21] Wu MS, Tan X, Huang JS, Wu JW, Jansson P (2015). Solute and water effects on soil freezing characteristics based on laboratory experiments. Cold Reg. Sci. Technol..

[22] Yi J (2014). Soil freezing and thawing processes affected by the different landscapes in the middle reaches of Heihe River Basin, Gansu, China. J. Hydrol..

[23] Jiang HB, Li L, Li ZQ (2020). Experimental study on channel water migration law and frost-heaving characteristics in seasonal frozen soil region. Water Resour. Hydropower Eng..

[24] Ma XS (2020). Analysis and simulation of soil moisture movement parameters during different freezing-thawing periods. J. Basic Sci. Eng..

[25] Nagare RM, Schincariol RA, Quinton WL, Hayashi M (2012). Effects of freezing on soil temperature, freezing front propagation and moisture redistribution in peat: laboratory investigations. Hydrol. Earth Syst. Sci..

[26] Teng K (1996). The regularity and development of groundwater in seasonal frozen soil area. Ground Water.

[27] Stahli M, Stadler D (1997). Measurement of water and solute dynamics in freezing soil columns with time domain reflectometry. J. Hydrol..

[28] Fang RL (1986). Preliminary study on the dynamical regime of water and salt during the freezing and thawing period of soil. Acta Pedol. Sin..

[29] Han LJ, Tsunekawa A, Tsubo M (2010). Monitoring near–surface soil freeze–thaw cycles in northern China and Mongolia from 1998 to 2007. Int. J. Appl. Earth Obs. Geoinf..

[30] Luo SS (2018). Aggregate–related changes in soil microbial communities under different ameliorant applications in saline–sodic soils. Geoderma.

[31] Yu PJ, Liu SW, Yang HT, Fan GH, Zhou DW (2018). Short-term land use conversions influence the profile distribution of soil salinity and sodicity in northeastern China. Ecol. Indic..

[32] Hinman UC (1970). Effects of freezing and thawing on some chemical properties of three soils. Can. J. Soil Sci..

[33] Li RR (2018). Effect of different vegetation restoration types on fundamental parameters, structural characteristics and the soil quality index of artificial soil. Soil Till. Res..

